# Collaboration of *Hprt/K-RAS/c-Myc* mutation in the oncogenesis of T-lymphocytic leukemia: a comparative study

**DOI:** 10.2144/fsoa-2023-0111

**Published:** 2024-05-15

**Authors:** Mai O Kadry, Abdel-Hamid Z Abdel Hamid, Rehab M Abdel-Megeed

**Affiliations:** 1 National Research Center, Therapeutic Chemistry Deparment, Al Bhoouth Street, Egypt

**Keywords:** *c-Myc*, DMBA, *Hprt*, *JAK2*, *K-RAS*, leukemia

## Abstract

**Aim:** Leukemia is a malignant clonal illness stem from the mutations of hematopoietic cells. Acute lymphoblastic leukemia is one of the utmost prevalent kinds of leukemia, is brought on by atypical lymphoid progenitor cell division in the bone marrow. **Materials & methods:** A comparative study between, titanium Nanoparticle-loaded doxorubicin or cisplatin and lactoferrin-loaded doxorubicin or cisplatin, on 7,12-dimethylbenz[a]-anthracene (DMBA)-induced leukemia was investigated and confirming the hypothesis that messenger RNA of *Hprt/K-RAS/c-Myc/SAT-2/P53/JAK-2* is a forthcoming signaling pathways in leukemia. **Results:** A significant alteration in *Hprt*, *K-RAS*, *C-Myc*, *P53*, *JAK-2* and *SAT-2* genes was observed post DMBA intoxication the aforementioned Nanodrugs modulated these signaling pathways. **Conclusion:** The carrier-loaded drugs triggered cytotoxicity of cancer cells via enhancing drug efficacy and bio-availability.

## Plain language summary

Leukemia is the abnormal growth of white blood cells that is responsible for fighting infection. Cisplatin and doxorubicin are commonly used anticancer drugs that can combat leukemic cells however they faced some problems of poor solubility and toxicity to normal cells. Thus we designed nanodrugs as Ti-NPs-cisplatin or DOX and lactoferrin-cisplatin or DOX and compared them with DOX and cisplatin and studied their impact on DMBA-induced leukemia in rat models. Monitoring apoptotic and cell survival genes was performed. Treatment with the nanodrugs could be promising in targeting cancer cells and improving drug bio-availability thus inducing cancer cell death.

## Abstract (plain language summary)

Induction of leukemia in rats via DMBA.

Treating leukemic rats with TiNPs-Dox or cisplatin and lactoferrin-Dox or cisplatin.

Monitoring *Hprt/K-RAS/c-Myc/SAT-2/P53/JAK-2* signaling pathways in leukemic rats.

The carrier-loaded drugs counteracted leukemia via triggering cytotoxicity of cancer cells and enhancing drug efficacy and bioavailability.

## Graphical abstract

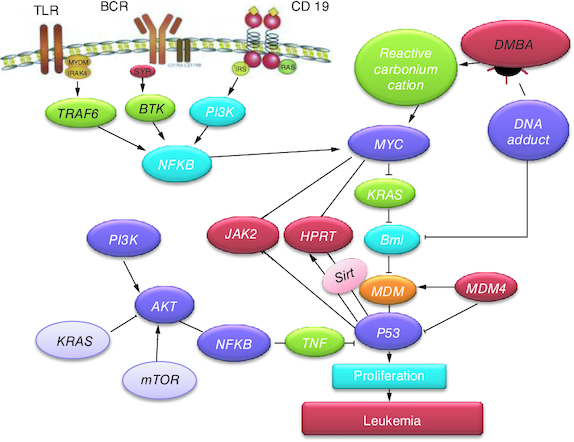

Cancer of the blood-generating tissues, such as the bone marrow and lymphatic system, is recognized as leukemia (T-ALL). There are several forms of leukemia. Leukemia often affects white blood cells, which are effective infection fighters, but in those suffering from the disease, the bone marrow overproduces aberrant white blood cells that don't function as they should [1]. Fever, bone pain, bleeding and elevated risk of infections is just a few of the leukemia symptoms. Leukemia stems from both genetic and environmental factors. Ionizing radiation, smoking and previous chemotherapy are risk factors [2,3]. Targeted therapy, chemotherapy, radiation therapy and bone marrow transplant are possible forms of treatment [4]. The most often utilized chemical carcinogen in animal models of leukemia is 7,12-dimethylbenz[a]-anthracene (DMBA). In addition to highlighting the DMBA-induced leukemia in animals, this article also discusses the roles played by related signaling pathways in the diagnosis of leukemia in laboratory animal's models and the subsequent development of therapies. Widespread evidence of *HPRT* mutation has been originated in leukemic patients. The *HPRT* alterations were characterized at the molecular level and categorized as either point mutations, such as single-nucleotide substitutions, deletions or insertions, or wide chromosomal rearrangements (GCRs) [5]. Remarkably, forced expression of *c-Myc* causes T-ALL [6] and protects a number of Notch1-dependent T-ALL cell lines from blockage of the Notch1 pathway [7]. The signaling threshold for *c-Myc* promoted via Notch1 seems to be comparatively elevated because L1601PP. In contrast, low levels of intracellular Notch1 (ICN1), such as those produced by the mutations L1594P and L1601P, seem to hasten T-ALL induction by potent oncogenes like *KRAS* [8]. Actually, when expressed in the presence of activated *KRAS*, even extremely weak Notch1 alleles, N1P, seem to produce a slight selection advantage. Other research teams have discovered that N1P alleles also augment T-ALL brought on by transgenic *c-Myc* and *E2a/Pbx* which is consistent with our findings [9,10]. DOX is a chemotherapeutic drug utilized in treating cancers such as T-ALL, lymphoma. Nausea, bone marrow suppression and oral irritation are typical adverse effects. It functions in part by interfering with DNA's ability to function [10]. There are also versions that are PEGylated and embedded in liposomes [11]. Multiple myeloma, ovarian and breast cancer are all indicated to be cured via doxorubicin PEGylated liposomal [12]. Via suppression and intercalation of the production of macromolecular, DOX interacts with DNA. This prevents topoisomerase II, an enzyme that loosens DNA super coils for transcription, from progressing [13]. DOX decreased the number of leukemia cells, caused the generation of spindle cells, increased cellular damage, and enhanced apoptosis. Cells treated with doxorubicin displayed signs of cell membrane rupture and loss of connection [14]. Several studies have previously discussed the role of *CIS* in treating specific forms of T-ALL [15]. It resulted in a complete remission in instances with ALL [16]. Novel techniques have spotted lights on targeted medicine transport to specific cells via nano biotechnology [17]. Due to its high potential as an alternate drug-delivery mechanism to traditional liposomes, titanium nanoformulations has recently been utilized as promising drug nanocarriers. They stand out for having a large interior surface area and being isotropic [18]. They are able to include various therapeutic compounds that exhibit lipophilic, hydrophilic or amphophilic properties. They are also biocompatible, bio-adhesive and biodegradable due to the lipid content [19].

The aim of the current study is to set a comparative study between loaded nanomedicine as titanium nanoformulation-loaded cisplatin and titanium nanoformulation-loaded doxorubicin with their non-loaded analogs (cisplatin and doxorubicin) and between lactoferrin-loaded doxorubicin and lactoferrin loaded-cisplatin with their non-loaded analogs and neupogen (standard drug) against DMBA-induced leukemia via monitoring the crosstalk between *Hprt/K-RAS/c-myc/SAT-2/P53/JAK-2*-signaling pathways.

## Materials & methods

### Chemicals

DMBA, doxorubicin, titanium-loaded doxorubicin, lactoferrin-loaded doxorubicin, cisplatin, titanium-loaded cisplatin, lactoferrin-loaded cisplatin and neupogen attained from Sigma-Aldrich Co. RT-PCR kits (*KRAS*, *HPrt*, *SAT-2* and *C-Myc*) were supplied from (Qiagen, USA). P53 and JAK-2 ELISA kits obtained from (R&D systems, MN, USA). Hb and Iron biomarkers were attained from Randox Company. Totally chemicals are of great analytical rank.

### Preparation of titanium-loaded doxorubicin

For standard drug loading, 1 ml of TiNPs (10 mg/ml) and 2 ml of DOX (2 mg/ml) in water were combined. To create DOX-TiNPs nanocomposites that serve as drug-delivery systems, the reaction mixture was left in the dark all night. By centrifuging at 5000× *g* for 20 min, DOX-TiNPs were isolated from the free-standing drug.

### Animals & treatments

Seventy two male western albino rats (170–190 g), from the animal house of National Research Center were utilized in the current investigation. The standard settings for animal care were 23 °C, 50% humidity. They possess admittance to water and a typical chow diet of pellets. Animal care and treatment protocols closely follow the ethical guidelines and rules that have been authorized by the US National Institutes of Health and the Animal Care and Use Committee of the National Research Center (19293).

### Experimental design

Animals were separated into nine groups of eight rats 1 week after acclimation, and were divided accordingly:

- Animals in (G1) acted as the control group and received saline;
- G2: received DMBA in a SC dose of (35 mg/kg body weight) every 7 days and served as a leukemia model [20]. Rats were kept for 3 months until the incidence of leukemia;
- G3: post leukemia induction treated with doxorubicin (5 mg/kg BW) IP for 1 month [14];
- G4: post leukemia induction treated via titanium-loaded doxorubicin (2 mg/kg BW) IP for 1 month [21];
- G5: post leukemia induction treated via lactoferrin-loaded doxorubicin (2 mg/kg BW) IP for 1 month [22];
- G6: following the induction of leukemia, cisplatin (5 mg/kg BW) IP was administered for 1 month [23];
- G7: post leukemia induction treated with titanium-loaded cisplatin (2 mg/kg BW IP) for 1 month [21];
- G8: post leukemia induction treated with lactoferrin-loaded cisplatin(2 mg/kg BW IP) for 1 month [22];
- G9: post leukemia induction treated with Neupogen (5 mg/kg BW IP) for 1 month [24].

### Blood sampling & tissue preparation

There rat's health was monitored. The animal's weight was scored, mildly sedated with carbon dioxide, and sublingual vein blood was taken. Sera was centrifuged at 3000 rpm for 15 min, and it was then stored at -80 °C for analysis of the biochemical and molecular analysis. Rats were sacrificed via cervical dislocation. Serum was divided into portions for biochemical and RT-PCR estimation.

### Measured biochemical parameters

#### Iron determination

Iron was estimated spectrophotometry via Randox Company kits according to manufacture instructions.

#### Hemoglobin determination

Hemoglobin was estimated spectrophotometry via Randox Company kits according to manufacture instructions.

#### mRNA gene expression of serum *Hprt*, *KRAS*, *SAT-2* & *c-Myc*

Using RT-PCR and particular forward and reverse primers for *Hprt*, *KRAS*, *SAT-2* and *c-Myc*, target gene expression analysis was examined (Table 1). Initially, total RNA was isolated from serum samples using the SV total RNA isolation system (Promega, WI, USA). Following reverse transcription into cDNA, the collected RNA will be amplified by PCR using the RT-PCR kit (Stratagene, CA, USA). The final volume for the reactions will be 50 l (25 l SYBR Green Mix [2×], 0.5 l cDNA, 2 l primer pair mix [5 pmol/leach primer], and 22.5 l water) [25].

**Table 1.** Primers sequence for RT-PCR analysis.

Primers	Sequence
Hprt1	Forward: 5′-GCTTCCTTCTCCGCAGACT-3′ Reverse: 5′-CTTCATCACGTCTCGAGCAA-3′
c-myc	Forward: 5′-ATCACAGCCCTCACTCAC-3′ Reverse: 5′-ACAGATTCCACAAGGTGC-3′
k-RAS	Forward: 5′-ATTATAAGGCCTGCTGAAAATGACTGA-3′ Reverse: 5′-ATATGCATATTAAAACAAGATTTACCTCTA-3′
β-actin	Forward: 5′-CTTTGATGTCACGCACGATTTC-3′ Reverse: 5′-GGGCCGCTCTAGGCACCAA-3′

### ELISA determination of P53 & JAK-2

The P53 and JAK-2 protein expression were determined using an enzyme-linked immunosorbent assay kit (R&D Systems, MN, USA), according to the manufacturer's instructions. Then quantitative sandwich enzyme immunoassay was used to evaluate the assay. Next, the micro plate was pre-coated with specific antibodies. Then, the immobilized antibody that bound to P53 and JAK-2 was added, and the wells were supplemented with the enzyme-linked secondary antibody specific for P53 and JAK-2, Afterward, the absorbance was determined at 450 nm [26].

### Statistical analysis

The results are expressed as mean ± SEM. Statistical analysis attained via the computer programmed Instat-3 (Graph pad software Inc, CA, USA). The SPSS 16 program was used to do a one-way analysis of variance (ANOVA), which was proceeded via *post hoc* Tukey's test when p ≤ 0.05.

## Results

### Inflection of leukemic biomarkers

**Figure 1.**
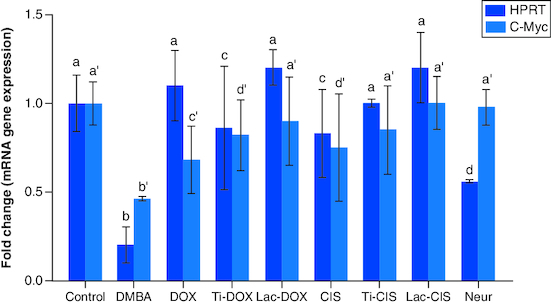
A comparative study between Ti-DOX, Lac-DOX, cisplatin, Ti-*CIS*, Lac-*CIS* and neupogen and their impact on *Hprt* and *C-Myc* gene expression post DMBA-induced leukemia. Data are expressed as mean ± S.E.M (n = 8). p ≤ 0.05 value is considered significant. Groups having the same letter are not significantly different from each other, while those having different letters are significantly different from each other. β-actin was used as reference gene.

**Figure 2.**
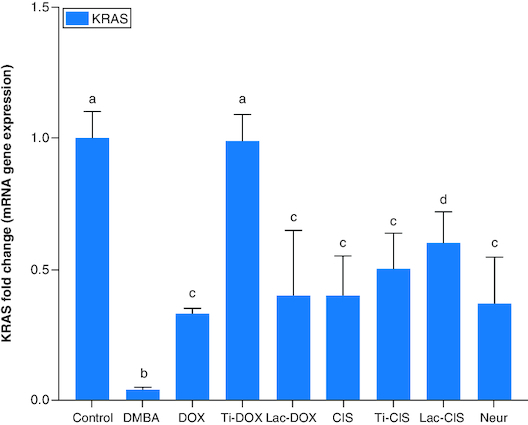
A comparative study between TiNPs-DOX, Lac-DOX, Cisplatin, TiNPs-*CIS*, Lac-*CIS* and Neupogen and their impact on *KRAS* gene expression post DMBA-induced leukemia. Data are expressed as mean ± S.E.M (n = 8). p ≤ 0.05 value is considered significant. Groups having the same letter are not significantly different from each other, while those having different letters are significantly different from each other. β-actin was used as reference gene.

**Figure 3.**
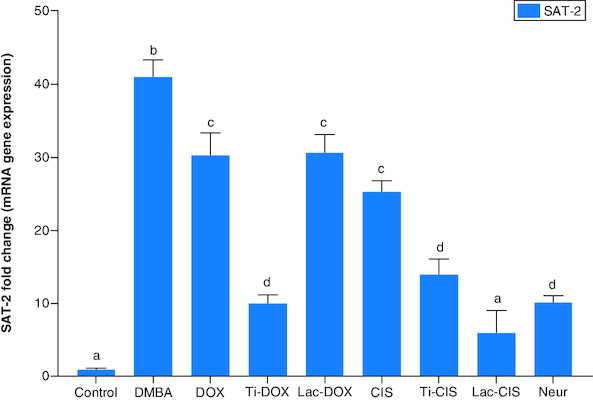
A comparative study between TiNPs-DOX, Lac-DOX, Cisplatin, TiNPs-*CIS*, Lac-*CIS* and Neupogen and their impact on *SAT-2* gene expression post DMBA-induced leukemia. Data are expressed as mean ± S.E.M (n = 8). p ≤ 0.05 value is considered significant. Groups having the same letter are not significantly different from each other, while those having different letters are significantly different from each other. β-actin was used as reference gene.

DMBA prompted a significant down regulation in *Hprt*, *KRAS* and *C-Myc* by about 0.2, 0.45 and 0.04 folds (Figures 1 & 2) as well as a significant up regulation in *SAT-2* gene expression by 41 folds (Figure 3). On the other hand, a concomitant modification in these parameters was noticed proceeding cisplatin, TiNPs-cisplatin and lactoferrin-cisplatin, doxorubicin, TiNPs-doxorubicin, lactoferrin–doxorubicin and neupogen treatment with the lactoferrin-loaded drugs elucidating the highest significant impact highlighting the impact of these gene mutations in T-ALL.

### Inflection of apoptotic biomarkers

**Figure 4.**
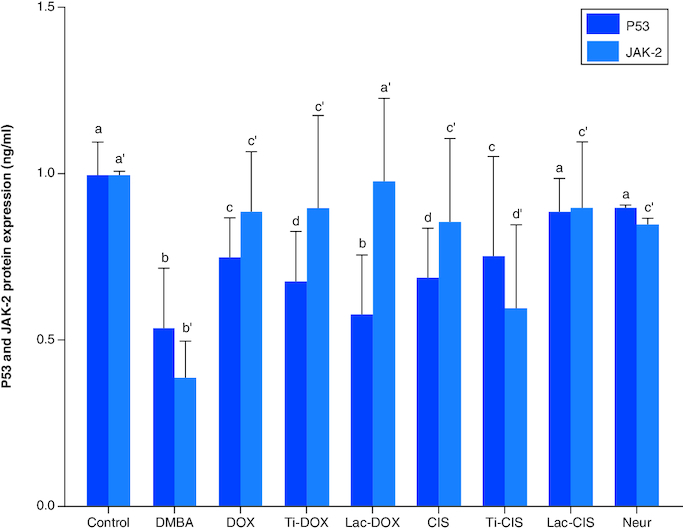
A comparative study between TiNPs-DOX, Lac-DOX, Cisplatin, TiNPs-*CIS*, Lac-*CIS* and Neupogen and their impact on P53 and JAK-2 protein expression post DMBA-induced leukemia. Data are expressed as mean ± S.E.M (n = 8). p ≤ 0.05 value is considered significant. Groups having the same letter are not significantly different from each other, while those having different letters are significantly different from each other.

DMBA prompted a significant down regulation in apoptotic biomarkers P53 and JAK-2 protein expression by about 0.54 and 0.4 folds. On the other hand, a concomitant modification in these parameters was noticed proceeding cisplatin, TiNPs-cisplatin and lactoferrin-cisplatin, Doxorubicin, TiNPs-doxorubicin, Lactoferrin-doxorubicin and Neupogen treatment with the lactoferrin-cisplatin and lactoferrin-doxorubicin elucidating the highest significant impact by about 0.89 and 0.98 folds, respectively, highlighting the impact of these genes mutation in T-ALL (Figure 4).

### Modulation of iron & hemoglobin levels

**Figure 5.**
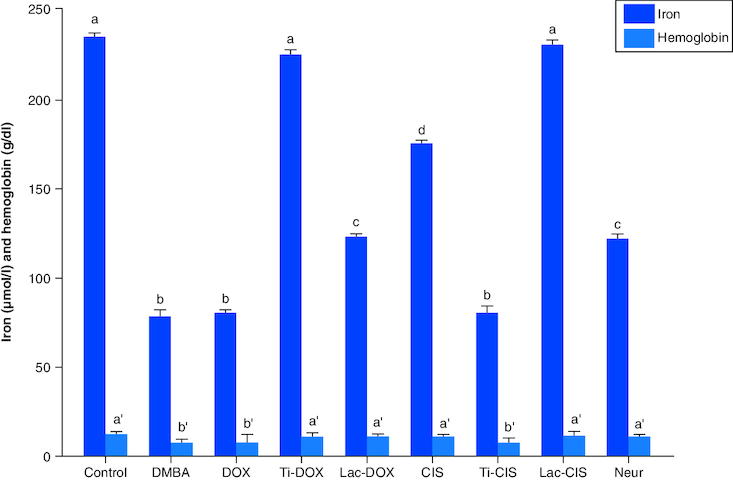
A comparative study between DOX, Ti-DOX, Lac-DOX, cisplatin, Ti-*CIS*, Lac-*CIS* and Neupogen and their impact on iron and hemoglobin levels post DMBA-induced leukemia. Data are expressed as mean ± S.E.M (n = 8). p ≤ 0.05 value is considered significant. Groups having the same letter are not significantly different from each other, while those having different letters are significantly different from each other.

**Figure 6.**
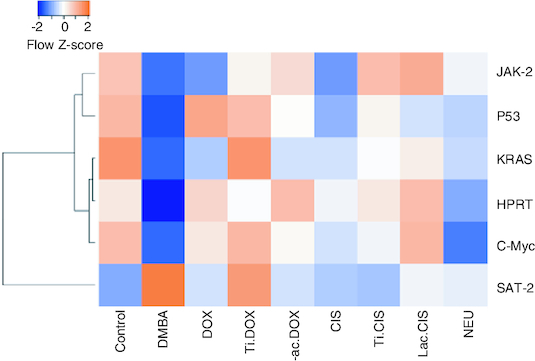
Heatmap representing different genes expression and their correlation. Orange represent high score while blue represent low score.

DMBA intoxication induced a significant reduction in both iron and Hb levels by a mean value of 78 and 7.8 of the normal value. However, a concomitant variation in these biomarkers was observed post Cisplatin, TiNPs-cisplatin and lactoferrin-cisplatin, doxorubicin, TiNPs-doxorubicin, lactoferrin-doxorubicin and Neupogen treatment with TiNPs-cisplatin and TiNPS-doxorubicin elucidating the most significant influence with a mean value of 11.5 and 12, respectively, reflecting the superiority of these drug-delivery systems in targeting cancer cells and reducing the disease impact (Figure 5). A heatmap was presented for different gene expression and their correlations (Figure 6).

## Discussion

Acute lymphoblastic leukemia (ALL) ranks second among adult acute leukemia in terms of prevalence. Despite the wide diversity of responses to chemotherapy, only 20–35% of patients accomplish long lasting recovery (Jones *et al.*, 2016). Novel approaches, such as liposomal-loaded medicine and nanoparticles that target specific organs without hurting healthy ones, are thus required to boost the rate of response, expand the period of recovery and decrease relapse.

The current study revealed a significant downregulation in *Hprt*, *KRAS* and *c-Myc* and a significant up regulation in *SAT-2* gene expression post DMBA intoxication. Meanwhile, they were modulated post DOX, TiNPs-DOX, LACT-DOX, *CIS*, TiNPs-*CIS*, LACT-*CIS* and Neupogen treatment. This is reflected by that elevated *c-Myc* is a mediator in *CIS* resistance via up regulating *cyclin E*. *c-Myc* is related to cell cycle progression via preventing p21 expression, a protein that dephosphorylates and tags Rb for degradation [27].

Human monocyte leukemia U937 cells readily undergo apoptosis when they are treated with TNF-α, anti-Fas antibody and anticancer drugs such as cisplatin and doxorubicin. To study the mechanism of apoptosis, a novel apoptosis-resistant variant, UC, from U937 cells was developed. The UC cells revealed resistance to apoptosis induced via TNF-α, anti-Fas antibody, cisplatin and doxorubicin. Mechanistic analysis revealed that UC cells expressed reduced amounts of *c-Myc*. This finding indicates that the reduced *c-Myc* expression in UC is strongly associated with the resistance to etoposide-induced apoptosis. Taken together, *c-Myc* plays a vital role in cellular susceptibility to death receptor-mediated and chemotherapy-induced apoptosis [28,29].

DNA adduct development is supposed to mediate the cytotoxic impact of cisplatin; however, these adducts may be responsible for mutagenic and secondary tumorigenic activities. In CHO-K1 cells, the *Hprt* gene was used to study the mutagenicity of cisplatin. Cisplatin caused dose-dependent increases in *Hprt* mutant frequency. Cisplatin caused a high frequency of G:C->A:T mutation and tandem base-pair substitutions primarily at positions 135 and 136 [30]. The majority of hereditary and somatic human mutations include G:C to A:T transitions, which has led to the theory that some of these mutations are caused by the synthesis of O^6^-methylguanine in DNA [31].

*KRAS* belongs to the *RAS* gene family that produces small G-proteins with intrinsic GTPase activity that inactivates proteins and regulates downstream effectors involved in differentiation, proliferation and apoptosis. Point mutations arise in tumors, which contribute to the loss of intrinsic GTPase activity and consequently in the deregulation of cell proliferation signals and increased aggressiveness of tumors. *KRAS* mutation in NSCLC reflects poor response to anticancer therapy and poor prognosis. In tumors, *KRAS* was detected to be mutated highly at codons 13 and 12 and a pool of mutations differing in the amino acid substitution and the base alteration. The DNA adduct formation and DNA damage responses implicated in cisplatin adducts removal revealed that the KRAS (G12C) mutation might be particular because it stimulates base excision repair to rapidly remove platinum from DNA even before the formation of cross-links [32]. A compromised response to DNA damage and mild G2/M phase arrest in *KRAS* (G12C) cells was discovered post cisplatin treatment [32].

The present article declared a significant reduction in P53 and JAK-2 protein expression post DMBA intoxication. Meanwhile, they were modulated post DOX, Ti-DOX, Lac-DOX, *CIS*, Ti-*CIS*, Lac-*CIS* and Neupogen treatment. This was reflected by that DMBA induced a rise in the phosphorylation of p53 and elevated p21 and PTEN and reduced both phosphorylated *c-Myc* and *c-Myc* mRNA levels [20]. DMBA reduced the expression of the onco/suppressor genes *c-Myc*, *KRAS* and *P53*, which are molecular epidemiological indicators of carcinogenesis. The conversion of DMBA into an active carbonium cation and subsequent alkylation of biological macromolecules, like DNA, constitute the DMBA mode of action.

Codon 61 mutation in the case of DMBA of *KRAS* gene was reported previously. It is the point mutation which is accountable for elevation in the *KRAS* gene, as well as *c-Myc* and *p53* genes which were distinguished post DMBA therapy in former *in vivo* researches. DMBA also reduced the expression of *Ha-RAS*, *p53* and *c-Myc* genes in the bone marrow. DMBA affects the expression pattern of the examined molecular epidemiological biomarker genes differently, despite the fact that a leukemic effect was observed for DMBA. In DMBA-induced bucal pouch carcinogenesis model, astaxanthin supplementation inhibits key *JAK-2/STAT* signaling pathways, particularly *STAT-3* phosphorylation and upregulates the expression of *SAT-2* target genes involved in cell proliferation, invasion and angiogenesis, as well as reduces vascular density, halting tumor progression [33].

For high-risk leukemia patients who are not resistant to targeted medicines, chemotherapy that contains cisplatin is a reasonable treatment option. When combined with chemotherapeutic medicines, *CIS* treatment had a greater cytotoxic effect on leukemia patients who had relapses. It revealed improvement, ranging from a significant decrease in bone marrow blasts to complete remission. One of the ways that *CIS* increases cytotoxicity is by inducing leukemic cell apoptosis at the mitochondrial level by changing the membrane potential of the mitochondria [34]. According to Wu *et al.* [34], *CIS* inhibits angiogenesis, shrinks tumors and motivates caspase cascade in various cancers. Leukemic cell lines' cell viability was reduced by *CIS* at low concentrations. The doses essential to limit cell survival within 60% are noticeably lower after *CIS* was included into Ti-NPs. NP compositions that are positively charged cause cellular penetration [15]. Apoptosis is one of the suggested machineries for the increased cell death. The annexin V/PI staining revealed that *CIS* titaniosomes had a greater percent of apoptotic cells than the medication alone. The mitochondrial membrane potential significantly decreased as a result of the TiNPs-*CIS* group, reflecting the initiation of mitochondrial respiratory arrest and death [15,35].

A cationic, amphipathic peptide called bovine lactoferricin (LfcinB) kills cancer cells in humans and rodents. Diverse leukemic and cancer cell lines quickly undergo apoptosis after receiving LfcinB therapy *in vitro*. The cytotoxic action of LfcinB was found to exist in FKCRRWQWRM amino acid sequence. When T-leukemia cells received LfcinB, ROS were produced, which then caused the mitochondrial *trans*-membrane potential to dissipate [36,37]. Caspase-9 and 3 were then activated, which cause apoptosis to cancer cells. Metastasis in the liver and lungs is significantly reduced after LfcinB administration. LfcinB has been shown to have an inhibitory impact on tumor growth and angiogenesis that have been injected with B16–BL6 melanoma cells. Furthermore, oral treatment with LfcinB in colon carcinogenesis causes a remarkable 85% decrease in the occurrence of colon adenocarcinoma. In addition, in cultures of THP-1 human monocytic leukemia cells, LfcinB is a strong promoter of apoptosis [22]. Ti-NPs, a type of nanoparticle utilized in combination therapy, have the ability to aggregate in the tumor, which makes it easier to distribute the medications where they are targeted. Moreover, co-encapsulation guarantees that the combined medications revealed nearly similar result [38,39]. Co-encapsulation further guarantees that the combined medications acquire more bioavailability and that their therapeutic index will be observed simultaneously by dose combination. Nanomedicines have the ability to prolong medication half-life in comparison to free pharmaceuticals, boosting drug accumulation in the tumor. And perhaps more crucially, drug release from nanocarriers can be adjusted. The medications should be promptly delivered into the tumor cells so as not to leak into the blood in order to reduce the negative side effects [35,40]. The ability of nanomedicines to assure a pharmacokinetic behavior of the co-encapsulated medications that is essentially equivalent is another major advantage. The DOX medication Vyxeos^®^ displayed identical pharmacokinetic profiles in the liposomal combination but exhibits significant differences when administered devoid of liposomes, is a perfect example [21].

## Conclusion

Titanium and lactoferrin-loaded nanomedicine could be a promising candidate for leukemia treatment via modifying that distinct *KRAS*, *Hprt*, *JAK2*, *P53*, *SAT-2* and *C-Myc* mutations and restoring hemoglobin and iron levels in DMBA induced leukemic rat model. Nanoparticle-loaded drugs could increase drug bioavailability, retention time and target organs.
